# Copy Number Alteration and Uniparental Disomy Analysis Categorizes Japanese Papillary Thyroid Carcinomas into Distinct Groups

**DOI:** 10.1371/journal.pone.0036063

**Published:** 2012-04-30

**Authors:** Michiko Matsuse, Kensaku Sasaki, Eijun Nishihara, Shigeki Minami, Chisa Hayashida, Hisayoshi Kondo, Keiji Suzuki, Vladimir Saenko, Koh-ichiro Yoshiura, Norisato Mitsutake, Shunichi Yamashita

**Affiliations:** 1 Department of Radiation Medical Sciences, Nagasaki University Graduate School of Biomedical Sciences, Nagasaki, Nagasaki, Japan; 2 Department of Human Genetics, Nagasaki University Graduate School of Biomedical Sciences, Nagasaki, Nagasaki, Japan; 3 Department of Internal Medicine, Kuma Hospital, Kobe, Hyogo, Japan; 4 Department of Surgery, Nagasaki University Graduate School of Biomedical Sciences, Nagasaki, Nagasaki, Japan; 5 Division of Scientific Data Registry, Atomic Bomb Disease Institute, Nagasaki University Graduate School of Biomedical Sciences, Nagasaki, Nagasaki, Japan; 6 Department of Health Risk Control, Nagasaki University Graduate School of Biomedical Sciences, Nagasaki, Nagasaki, Japan; 7 Nagasaki University Research Centre for Genomic Instability and Carcinogenesis (NRGIC), Nagasaki, Nagasaki, Japan; Consiglio Nazionale delle Ricerche (CNR), Italy

## Abstract

The aim of the present study was to investigate chromosomal aberrations in sporadic Japanese papillary thyroid carcinomas (PTCs), concomitant with the analysis of oncogene mutational status. Twenty-five PTCs (11 with *BRAF^V600E^*, 4 with *RET/PTC1*, and 10 without mutation in *HRAS*, *KRAS*, *NRAS*, *BRAF*, *RET/PTC1*, or *RET/PTC3*) were analyzed using Genome-Wide Human SNP Array 6.0 which allows us to detect copy number alteration (CNA) and uniparental disomy (UPD), also referred to as copy neutral loss of heterozygosity, in a single experiment. The Japanese PTCs showed relatively stable karyotypes. Seven cases (28%) showed CNA(s), and 6 (24%) showed UPD(s). Interestingly, CNA and UPD were rarely overlapped in the same tumor; the only one advanced case showed both CNA and UPD with a highly complex karyotype. Thirteen (52%) showed neither CNA nor UPD. Regarding CNA, deletions tended to be more frequent than amplifications. The most frequent and recurrent region was the deletion in chromosome 22; however, it was found in only 4 cases (16%). The degree of genomic instability did not depend on the oncogene status. However, in oncogene-positive cases (*BRAF^V600E^* and *RET/PTC1*), tumors with CNA/UPD were less frequent (5/15, 33%), whereas tumors with CNA/UPD were more frequent in oncogene-negative cases (7/10, 70%), suggesting that chromosomal aberrations may play a role in the development of PTC, especially in oncogene-negative tumors. These data suggest that Japanese PTCs may be classified into three distinct groups: CNA^+^, UPD^+^, and no chromosomal aberrations. *BRAF^V600E^* mutational status did not correlate with any parameters of chromosomal defects.

## Introduction

Papillary thyroid carcinoma (PTC) is the most common malignant tumor in endocrine organs, and its incidence has increased over the past decades [1]. PTCs have characteristic genetic alterations leading to the activation of mitogen-activated protein kinase (MAPK) signaling pathway. Those include *RET/PTC* rearrangements and point mutations in *BRAF* and *RAS* family genes [2]. They are found in approximately 60–70% of all PTCs and rarely overlap in the same tumor [3]–[5]. The lack of coexistence provides strong genetic evidence for the requirement of constitutively active MAPK signaling for development of PTC. However, the remaining 30–40% of PTC cases do not have genetic changes in these genes, suggesting that other factors may exist as a trigger to activate the MAPK pathway.

The *BRAF* mutation is the most prevalent genetic alteration in PTCs (44% on average) [1]. The mutation in PTCs is almost exclusively a thymine-to-adenine transversion at nucleotide 1799 in exon 15, resulting in a valine-to-glutamic acid substitution at amino acid 600 (V600E) [1]. This mutation is believed to produce a constitutively active kinase by disrupting hydrophobic interactions between residues in the activation loop and residues in the ATP binding site, allowing development of new interaction that fold the kinase into a catalytically competent structure [6].

Many studies, but not all of them, have demonstrated the associations between this mutation and clinicopathological aggressiveness such as extrathyroidal invasion, lymph node metastasis, advanced stage, and recurrence [1]. Indeed, transgenic mice overexpressing BRAF^V600E^ in thyroid cells develop invasive PTCs with some tall-cell features and poorly differentiated areas [7]. In addition, conditional expression of BRAF^V600E^ in the rat normal thyroid PCCL3 cells induced invasion and chromosomal instability, which was not observed in the same system with RET/PTC oncoprotein [8], [9].

Chromosomal aberrations have been reported at relatively low frequency in PTCs [10]–[17]. In these reports, comparative genomic hybridization (CGH) which provides a low resolution was used. However, two studies using array CGH, which enables a higher resolution depending on the number of probes, demonstrated a wide range of chromosomal aberrations [18], [19]. In contrast to CGH or array CGH, single nucleotide polymorphism array (SNP-A) as a molecular karyotyping tool has excellent resolution and allows us to detect copy number alteration (CNA) and also uniparental disomy (UPD) in the same experiment. The detection of UPD, also referred to as copy neutral loss of heterozygosity (CN-LOH), along whole genome is one of the advantages of SNP-A. The discovery of UPD in cancers has indicated that LOH is not necessarily due to loss of chromosomal material. In regions of UPD, portions of one of the chromosomes are lost and replaced by the exact copy of the other remaining chromosome, resulting in retention of two copies of genetic information but loss of polymorphic differences (both are from the same parent), which can be detected by SNP-A but not by conventional cytogenetic analysis such as CGH. UPD has been reported to be associated with both oncogenic mutations and inactivation of tumor suppressor genes in cancers [20]. UPD may be an important mechanism in cancer development [21], [22]. To the best of our knowledge, there has been only one study using SNP-A for PTC; 10 cases of pediatric radiation-associated post-Chernobyl PTCs were analyzed using Affymetrix 50 K Mapping array [23]. However, no significant regions of LOH were identified in this analysis. General sporadic PTCs have not been analyzed using SNP-A.

In the present study, we analyzed 25 Japanese sporadic PTCs with or without *BRAF^V600E^* mutation using Affymetrix Genome-Wide Human SNP Array 6.0 (SNP6.0) which gives much higher resolution (1.8 M probes) compared to the previous studies. This is the first report showing the presence of UPD in PTCs. We aimed to assess whether *BRAF* mutational status is associated with genomic instability and characteristic chromosomal aberrations represented by CNA and UPD. We also attempted to identify novel chromosomal portions that may contain genes involved in PTC pathogenesis.

## Materials and Methods

### Patients and Tumor Tissues

Fresh tumor tissue samples were collected from 25 patients with sporadic PTC (mean±SD age 53.3±16.3 years old, range 18–81 years old; 84.0% women; no any pretreatment), snap-frozen in liquid nitrogen, and stored at −80°C until use. An appropriate written informed consent was obtained from each patient, and the study was approved by the ethics committees of Nagasaki University and Kuma Hospital. All patients had no history of radiation exposure. Clinical and pathological characteristics of the patients including UICC TNM staging are listed in Table 1. Among 25 PTC samples, 11 had *BRAF^V600E^* mutation, 4 had *RET/PTC1* rearrangement, and 10 did not have any of the following oncogenic alterations: *HRAS*, *KRAS*, *NRAS*, and *BRAF* mutations; and *RET/PTC1* and *RET/PTC3* rearrangements. In this study, no new sequence data was generated.

**Table 1:** Clinicopthological data and genomic alterations in the 25 Japanese PTC cases.

Case	BRAF*^V600E^*	RET/PTC1 rearrangement	CNA	AS-UPD(CN-LOH)		Percent of altered genome	Age	Sex	Tumor size (mm)	Histological type	pEx	pT	pN	M	Vessel invasion	Stage	Recurrence
T13	+	−	−	+	0.5		45	F	23	p	0	1b–m	1a	0	−	III	−
T15	+	−	−	−	0.0		37	F	35	p	2	4a	1a	0	−	I	−
T17	+	−	+	+	16.8		75	F	53	p+poorly	2	4a	1b	M1–lung	v+++	IVC	+
T21	+	−	+	−	1.2		63	F	23	p	1	3	x	0	−	III	−
T23	+	−	−	+	2.3		62	F	38	p	2	4a	1b	0	−	IVA	−
T24	+	−	−	−	0.0		60	F	22	p	1	3	0	0	−	III	−
T25	+	−	−	−	0.0		36	F	27	p	1	3	0	0	−	I	−
T30	+	−	−	−	0.0		74	F	39	p	1	3	0	0	−	III	−
T45	+	−	−	−	0.0		69	F	36	p+poorly	1	3	1a	0	−	III	−
T53	+	−	−	−	0.0		60	M	20	p	1	3	1a	0	ND	III	−
T58	+	−	−	−	0.0		46	F	29	p	x	x	1b	0	−	IVA	−
T08	−	+	−	−	0.0		22	F	15	p	1	3	1b	0	v+, ly+	I	−
T18	−	+	−	−	0.0		18	F	33	p	1	3	1b	0	−	I	−
T19	−	+	+	−	1.8		36	F	20	p	0	1b	x	0	−	I	−
T20	−	+	−	−	0.0		41	F	12	p	0	1b	1b	0	−	I	−
T05	−	−	−	+	2.0		70	F	36	p	0	2	1b	0	ND	IVA	−
T06	−	−	−	+	6.0		42	F	24	fv	0	2	0	0	ND	I	−
T07	−	−	−	+	2.3		46	F	8	p	1	3	x	0	ND	III	−
T11	−	−	−	−	0.0		63	F	37	p	1	3	1a	0	ND	III	−
T27	−	−	−	−	0.0		49	F	24	p	0	1b	1b	0	ND	IVA	−
T32	−	−	+	−	1.7		56	M	45	fv	0	3	1b	0	ND	IVA	−
T36	−	−	+	−	0.3		58	M	20	fv	1	3	1b	0	ND	IVA	−
T47	−	−	−	−	0.0		68	F	20	p	0	1b	1a	0	ND	III	−
T48	−	−	+	−	1.2		81	F	25	p	1	3	x	0	ND	III	−
T50	−	−	+	−	3.7		56	M	65	p+poorly	1	3	1a	0	−	III	−

Using the UICC TNM classification.

Other abbreviations, F: female; M: male; p: papillary; fvp: follicular variant; ND: not described.

### Genomic DNA Extraction and Mutation Screening for BRAF and RAS

DNA was extracted from the frozen tissues using QIAamp DNA mini kit (QIAGEN, Tokyo, Japan) according to the manufacturer’s protocol. Common mutational hotspots of *BRAF* (exon 15) and *HRAS*, *KRAS* and *NRAS* (codons 12, 13, and 61) were examined by direct DNA sequencing. Primer sequences used for PCR and sequencing are listed in Table S1. PCR amplification was done using AmpliTaq Gold (Applied Biosystems, Life Technologies Japan, Tokyo, Japan). PCR products were treated with ExoSAP-IT PCR clean-up reagent (GE Healthcare Japan, Tokyo, Japan), and sequencing was performed with Big Dye Terminator sequencing kit version 3.1 (Applied Biosystems) on an ABI3100 automated sequencer (Applied Biosystems).

### RET/PTC Rearrangement Screening

For *RET/PTC* screening, RNA extraction and RT-PCR were done. Briefly, total RNA was extracted from the frozen tissues using ISOGEN (Nippon Gene, Tokyo, Japan) reagent according to the manufacturer’s instruction. The RNA was then reverse transcribed using MMLV-RT (Applied Biosystems) and random hexamers to generate cDNA. Subsequent PCR amplification was performed using AmpliTaq Gold. Primer sequences are listed in Table S1.

### Copy Number Detection and SNP Genotyping by High Resolution Genome-wide DNA Microarray

We performed high-resolution genome-wide DNA copy number detection and single nucleotide polymorphism (SNP) genotyping using Genome-Wide Human SNP Array 6.0 (SNP6.0) that interrogates 906,600 SNPs and 945,826 copy number probes, following the manufacturer’s instructions (Affymetrix Japan, Tokyo, Japan). Array scanning and genotyping were performed using Affymetrix GeneChip Command Console (AGCC) and Birdseed v2 algorithm of Genotyping Console (GTC) 4.0 software (Affymetrix). As a quality control of the genotyping, Contrast QC value was calculated as implemented in the GTC 4.0. Genomic positions of the SNPs corresponded to the March 2006 human genome (hg18). Copy number and allele ratio analysis was performed by Partek Genomics Suite version 6.5 (Partek Inc., St. Louis, MO, USA). Reference generated from the intensities of pooled 52 normal Japanese sample profiles in our laboratory was used. The data sets were also used as a baseline to determine LOH. Genomic segmentation method was used to detect amplified or deleted segments using Partek Genomics Suite with stringent parameters (p≤0.0001, ≥20 markers, signal/noise ≥0.6), and only segments ≥100 probes were considered for further analysis to avoid false positive [24].

UPD areas were also confirmed using Hidden Markov Model and Copy Number Analyser for GeneChip 3.0/Allele-specific Copy Number Analysis using anonymous References (CNAG/AsCNAR) as previously described (compared with 3–10 best fit normal references with the lowest SD) [25], [26]. Copy number variations (CNVs) seen in both our SNP6.0 analysis and the Database of Genomic Variants (DGV, http://projects.tcag.ca/variation/) were considered as tumor-unrelated CNVs and excluded from further analysis [27]. When run of homozygosity (ROH) was detected, telomeric lesions or interstitial defects ≥24.6 mega bases (Mb) were considered true acquired somatic UPD (AS-UPD), since small region of homozygosity can result from autozygosity or germline UPD [27]–[29]. In a strict sense, however, AS-UPD (or CN-LOH) can only be confirmed by comparing tumor tissue with non-tumor tissue in one individual. Accordingly, the term “UPD” used in this study is rigorously equal to a relatively large segment region of ROH, although it is most likely AS-UPD.

Regions of CNA or UPD were cross-referenced with Refseq genes (http://www.ncbi.nlm.nih.gov/RefSeq/) and the CancerGene database (http://cbio.mskcc.org/CancerGenes/Select.action) [30].

### Comparison of Genomic Instability and Statistical Analysis

To estimate genomic instability, the length of each genomic alteration (CNA and UPD) was summed, and the total length was divided by 2,858,034,764 bases, which is the total length of human genome (hg18), to calculate the percentage of altered genome.

Differences between groups divided by the status of oncogenic mutations were examined for statistical significance with Kruskal-Wallis test.

Odds ratios and 95% confidence intervals for the association between the presence of chromosomal aberration(s) and clinicopathological parameters including *BRAF* mutational status were estimated using unconditional logistic regression models including age, tumor size, histological type, and stage.

## Results

### CNA and UPD in the Japanese PTCs

**Figure 1:**
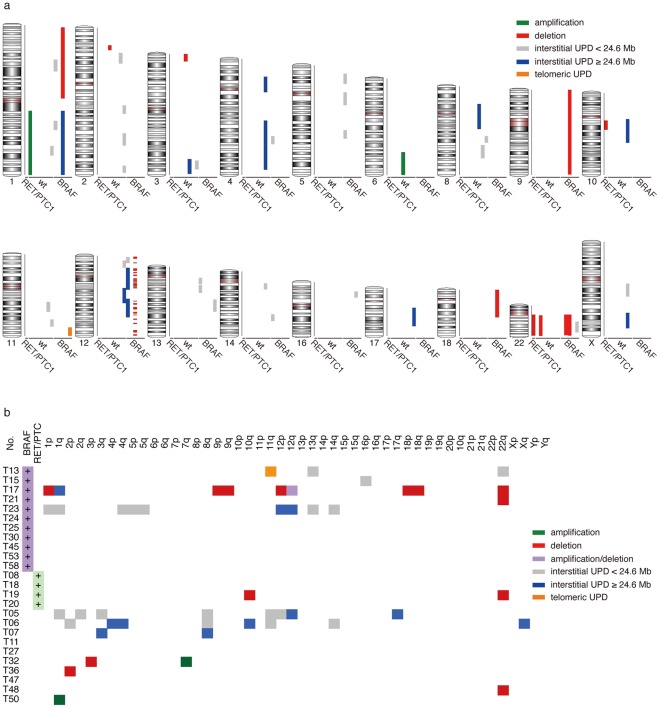
Genome-wide view of detected CNA and UPD. Twenty-five PTCs (11 with *BRAF^V600E^*, 4 with *RET/PTC1*, and 10 without mutation in *HRAS*, *KRAS*, *NRAS*, *BRAF*, *RET/PTC1*, or *RET/PTC3*) were analyzed using Genome-Wide Human SNP Array 6.0. Green lines: amplifications, red lines: deletions, blue lines: large (≥24.6 Mb) interstitial UPD regions, gray lines: small interstitial UPD regions (at least 10 Mb but no more than 24.6 Mb), orange lines: telomeric regions of UPD. a, Chromosomal view. Each line represents an individual aberration. The extent of the aberration is represented along the length of the chromosome. b, Case (oncogene status)-chromosome plot, which provides at-a-glance view of all aberrations in a single case. The extent of the aberrations is ignored.

We analyzed genomic DNA from 25 Japanese PTC tissues by SNP6.0. A genome-wide view of detected CNA and UPD is shown in Figure 1 and summarized in Table S2. UPDs >10 Mb and <24.6 Mb were also included for reference although we cannot exclude the possibility that these UPDs are germline. However, all these short UPDs were observed along with long UPDs (≥24.6 Mb) in all the cases except one (T15, Figure 1b), suggesting that most of these short UPDs are likely to be acquired somatic UPD (AS-UPD) (see Materials and Methods section). Nevertheless, the short UPDs were excluded from the further analyses due to uncertainty.

Japanese PTC samples are very heterogeneous concerning the number, size, and location of CNA/UPD. The majority of the cases showed a few or no CNA/UPD (Figure 1b), and interestingly, CNA and UPD were rarely overlapped in the same tumor. Seven cases (T17, T19, T21, T32, T36, T48, T50; 28%) showed CNA(s), and 6 (T05, T06, T07, T13, T17, T23; 24%) showed UPD(s) (Table S2, Figure S1, and S2). Only one case (T17; 4%) showed both CNA and UPD with a highly complex karyotype (Table S2 and Figure S1). Thirteen (T08, T11, T15, T18, T20, T24, T25, T27, T30, T45, T47, T53, T58; 52%) showed neither CNA nor UPD. Regarding CNA, deletions tended to be more frequent than amplifications (Figure 1). Associations between CNA (deletion and amplification)/UPD and clinicopathological parameters including *BRAF* mutational status were estimated using unconditional logistic regression models. Only follicular variant (fv) histotype (vs. classic papillary) was weakly associated with deletion (Odds ratio = 15.6, 95% confidence interval: 0.75–795.4, p = 0.098) but note that only three fv cases were included in our analysis. No obvious correlation was found between chromosomal aberrations and any other parameters (Table 1).

### Comparison of the Japanese PTCs by the Status of Oncogenic Mutation

CNA occurred in 2 PTCs with *BRAF^V600E^* (2/11, 18%), 1 with *RET/PTC1* (1/4, 25%), and 4 without oncogenic mutation (no *HRAS*, *NRAS*, *KRAS*, *BRAF^V600E^*, *RET/PTC1*, *RET/PTC3*) (4/10, 40%) (Figure 1 and Table 1). The most frequent and recurrent CNA was the deletion in chromosome 22; however, it was found in only 4 cases (4/25, 16%): 2 PTCs with *BRAF^V600E^*, 1 with *RET/PTC1*, and 1 with no mutation (Figure 1). No specific CNA was found in each oncogene group (Figure 1).

UPD was found in 3 PTCs with BRAF^V600E^ (3/11, 27%) and in 3 with no mutation (3/10, 30%) (Figure 1 and Table 1). We found 11 interstitial UPDs (range, 24.6–105.7 Mb) and 1 UPD extending to the telomere. These UPDs were not overlapped, except for small regions on chromosome 12q (T23 and T05) (Figure 1 and Figure S2).

Interestingly, in oncogene-positive cases (*BRAF^V600E^* and *RET/PTC1*), tumors with CNA/UPD were less frequent (5/15, 33%), whereas tumors with CNA/UPD were more frequent in oncogene-negative cases (7/10, 70%).

**Figure 2:**
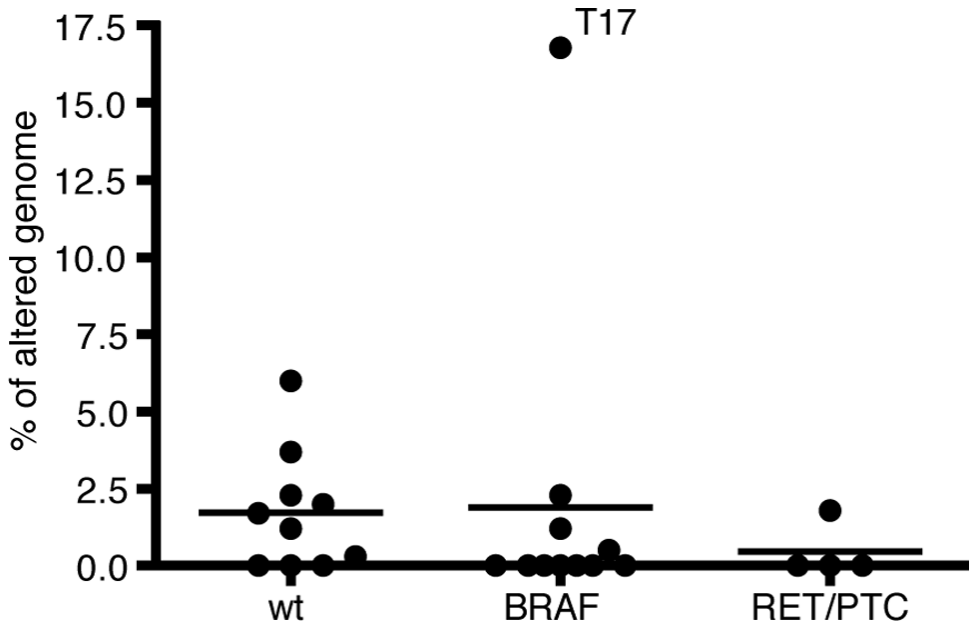
The degree of genomic instability among three oncogene groups (wild-type, *BRAF^V600E^*, and *RET/PTC1*). Each dot represents the percentage of altered genome of each case. The length of each genomic alteration (CNA/UPD) was summed, and the total length was divided by 2,858,034,764 bases to calculate the percentage of altered genome. Horizontal line shows mean. T17 case had a highly complex karyotype and was the only case with distant metastasis/recurrence. There was no statistical difference among these groups.

Next, we also measured the degree of genomic instability using the percentage of altered genome (Table 1). As shown in Figure 2, there was no statistical difference among three oncogene groups (no mutation, *BRAF^V600E^*, and *RET/PTC1*).

### Candidate Genes Involved in the Japanese PTC Pathogenesis

To find important tumorigenic events in PTC, we searched for CNA/UPD that was commonly shared among the samples. As mentioned above, the only recurrent genomic aberration was the deletion on chromosome 22q in 4 samples (4/25, 16%), which was independent of the oncogene status (Figure 1). The 22q deletion was large, spanning a 34 Mb region and containing 66 genes registered at the CancerGenes database (Table S3), some of which are potential tumor suppressor genes (*TOP3B*, *CHEK2*, *NF2*, *LIF*, *DUSP18*, *TIMP3*, *XRCC6*, and *PPARA*).

Since constitutive activation of the MAPK pathway is thought to be critical for PTC development, we next searched for RAS family/MAPK pathway genes (at CancerGenes) and also PTC-related genes reported in literature within CNA/UPD regions, regardless of their frequency. Selected candidate genes are listed in Table S4.

## Discussion

In the present study, we have performed SNP6.0 analyses using the Japanese PTC tissue samples. As far as we know, this is the first report using SNP6.0 for molecular karyotyping of PTCs and also demonstrating the occurrence of UPD. SNP6.0 contains 1.8 M probes and achieves ultra high resolution (1 copy number marker every 700 bp on average). Since this study is unpaired analysis, we used stringent parameters to avoid false positive and also the CNAG/AsCNAR algorithm (see Materials and Methods section) which enables accurate determination of allele-specific copy number without using paired normal DNA as well as the sensitive detection of LOH (including UPD) even in the presence of up to 70–80% of normal cell contamination. Moreover, we also utilized public copy number variation (CNV) database because CNVs are common in a wide range of human individual genome, covering 1002 Mb (35% of the genome) (http://projects.tcag.ca/variation/). Indeed, we excluded most of the small regions that were overlapped with the CNVs in the database. We believe that we avoided false positive but probably had false negative areas, although they were small regions.

Of 25 PTC cases, our data demonstrate relatively stable karyotypes: 7 CNAs, 6 UPDs (one case has both CNAs and UPD), and 13 normal karyotypes, consistent with earlier studies using conventional CGH or low resolution array CGH [10]–[17]. However, two recent studies using higher resolution array CGH and SNP-A reported chromosomal aberrations in all analyzed samples [18], [23]. Although CNV was not taken into consideration in these analyses, there were obvious differences among 3 studies including ours. Unger et al. demonstrated nearly whole loss of chromosome 17, 19, and 22 in most of adult *RET/PTC*-negative cases, which is not consistent with our data, and also loss of chromosome 6, 9p, and 13 in childhood cases, which is not matched to the study by Stein et al. We do not have any feasible explanation to solve this discrepancy. This might be due to ethnic variations.

Japanese PTCs have some distinct characteristics: the vast majority are low risk tumors with classic papillary morphology and higher prevalence of the *BRAF* mutation than in PTCs in western countries [31]–[34]. These characteristics are presumably due to high iodine intake in Japanese population [35]–[37]. There have been a large number of publications reporting the relationship between *BRAF^V600E^* mutation and clinicopathological aggressiveness (reviewed in [1]). *In vivo* and *in vitro* studies have also indicated that *BRAF^V600E^* drives aggressiveness and genomic instability [8], [9]. However, a recent study analyzing more than 600 Japanese patients in a single institution has demonstrated extremely good prognosis and no correlation of the *BRAF* mutational status with clinicopathological aggressiveness [38]. This is in line with our results: low prevalence of CNA/UPD and no relationship between *BRAF* status and genomic instability in the Japanese PTCs. Further studies using samples from different ethnicities are needed.

It is of great interest that CNA and UPD were rarely overlapped in the same tumor. Five tumors with UPD had no CNA; six tumors with CNA had no UPD; only one tumor, T17 had both CNA and UPD. T17 was the only case with distant metastasis (lung), prominent vascular invasion, and recurrence. Along with highly complicated genomic aberrations, T17 is the end stage of the disease and may be exceptional. Thus, Japanese PTCs may be classified into three distinct groups: CNA^+^, UPD^+^, and no chromosomal aberrations, suggesting that different ways of acquiring genomic instability exist in PTCs. The frequency of CNA/UPD was higher in oncogene-negative cases (70%) than in oncogene-positive cases (33%), suggesting that chromosomal aberrations (or affected gene sets) may play a role in the development of PTC, especially in oncogene-negative tumors, albeit not statistically significant (Fisher’s exact test, odds ratio = 4.667, 95% confidence interval: 0.83–26.25, p = 0.11). This implies that there may be distinct mechanisms for PTC development through oncogenic mutation and/or chromosomal instability.

We tried to identify novel chromosomal regions/genes involved in PTC pathogenesis, depending on mutational status of the oncogenes. However, the pattern of CNA/UPD in the Japanese cases was very heterogenous. We only found the 22q deletion as a recurrent region, which is consistent with previous studies [12]–[17]. However, the 22q deletion was found in only 4 cases, suggesting that it may be difficult to find specific chromosomal aberrations that contain responsible genes for PTC development even though SNP6.0 was utilized. Furthermore, the presence of the 22q deletion was independent of the oncogene status. We selected candidate genes from the 22q area and other CNA/UPD regions using the CancerGenes database. Among the selected genes in the 22q area, *CHEK2* [39], *LIMK2* [40], and *TIMP3* [41] have already been reported to be deleted/down-regulated in thyroid cancer; the 22q deletion may play a role in thyroid carcinogenesis. Regarding the selected genes in other CNA/UPD areas, the *AKT3*/*PIK3CA* pathway [42], [43], the *KRAS*/*RAP1B*/*RAP1GAP*/*BRAF* pathway [44], [45], and the *VEGFC* pathway [46], [47] have been known to participate in thyroid carcinogenesis/cancer progression. However, none of them is universal in PTCs.

In conclusion, we report for the first time the results of genome-wide molecular karyotyping using SNP6.0 concomitant with the analysis of oncogene mutational status in PTCs. The Japanese PTCs showed relatively stable karyotypes: approximately 1/4 of CNA, 1/4 of UPD, and 1/2 of no aberrations. We also demonstrate for the first time that UPD as a form of genomic instability may play a role in PTC development.

## Supporting Information

Figure S1 **CNA regions identified using PartekGS software.** CN: smoothed copy number is plotted, AsCN: allele-specific copy number analysis in which each allele is plotted separately, AR: allele ratio pattern.
(PDF)

Figure S2 **Whole-genome view of 5 PTC cases with UPD generated by CNAG/AsCNAR.** CN: linear copy number smoothed over 10 SNPs is plotted in chromosomal order along the horizontal axis (chromosome 1 on the left, chromosome 22 on the right), Hetero: green bars indicate heterozygous call, AsCN: allele-specific copy number analysis in which each allele is plotted separately. Blue box represents interstitial UPD (≥24.6 Mb); Orange box represents telomeric UPD.
(PDF)

Table S1 Primer sequences.
(PDF)

Table S2 Chromosomal aberrations in Japanese PTCs.
(PDF)

Table S3 CancerGenes located in recurrent chromosome 22q deletion.
(PDF)

Table S4 RAS family/MAPK pathway genes from CancerGenes database and PTC-related genes located in the CNA/UPD region.
(PDF)

## References

[1] Xing M (2007). BRAF mutation in papillary thyroid cancer: pathogenic role, molecular bases, and clinical implications.. Endocr Rev.

[2] Kondo T, Ezzat S, Asa SL (2006). Pathogenetic mechanisms in thyroid follicular-cell neoplasia.. Nat Rev Cancer.

[3] Frattini M, Ferrario C, Bressan P, Balestra D, De Cecco L (2004). Alternative mutations of BRAF, RET and NTRK1 are associated with similar but distinct gene expression patterns in papillary thyroid cancer.. Oncogene.

[4] Kimura ET, Nikiforova MN, Zhu Z, Knauf JA, Nikiforov YE (2003). High prevalence of BRAF mutations in thyroid cancer: genetic evidence for constitutive activation of the RET/PTC-RAS-BRAF signaling pathway in papillary thyroid carcinoma.. Cancer Res.

[5] Soares P, Trovisco V, Rocha AS, Lima J, Castro P (2003). BRAF mutations and RET/PTC rearrangements are alternative events in the etiopathogenesis of PTC.. Oncogene.

[6] Wan PT, Garnett MJ, Roe SM, Lee S, Niculescu-Duvaz D (2004). Mechanism of activation of the RAF-ERK signaling pathway by oncogenic mutations of B-RAF.. Cell.

[7] Knauf JA, Ma X, Smith EP, Zhang L, Mitsutake N (2005). Targeted expression of BRAFV600E in thyroid cells of transgenic mice results in papillary thyroid cancers that undergo dedifferentiation.. Cancer Res.

[8] Mitsutake N, Knauf JA, Mitsutake S, Mesa C, Zhang L (2005). Conditional BRAFV600E expression induces DNA synthesis, apoptosis, dedifferentiation, and chromosomal instability in thyroid PCCL3 cells.. Cancer Res.

[9] Mesa C, Mirza M, Mitsutake N, Sartor M, Medvedovic M (2006). Conditional activation of RET/PTC3 and BRAFV600E in thyroid cells is associated with gene expression profiles that predict a preferential role of BRAF in extracellular matrix remodeling.. Cancer Res.

[10] Bauer AJ, Cavalli LR, Rone JD, Francis GL, Burch HB (2002). Evaluation of adult papillary thyroid carcinomas by comparative genomic hybridization and microsatellite instability analysis.. Cancer Genet Cytogenet.

[11] Hemmer S, Wasenius VM, Knuutila S, Franssila K, Joensuu H (1999). DNA copy number changes in thyroid carcinoma.. Am J Pathol.

[12] Singh B, Lim D, Cigudosa JC, Ghossein R, Shaha AR (2000). Screening for genetic aberrations in papillary thyroid cancer by using comparative genomic hybridization.. Surgery 128: 888–893;discussion.

[13] Wreesmann VB, Ghossein RA, Hezel M, Banerjee D, Shaha AR (2004). Follicular variant of papillary thyroid carcinoma: genome-wide appraisal of a controversial entity.. Genes Chromosomes Cancer.

[14] Richter H, Braselmann H, Hieber L, Thomas G, Bogdanova T (2004). Chromosomal imbalances in post-chernobyl thyroid tumors.. Thyroid.

[15] Rodrigues R, Roque L, Espadinha C, Pinto A, Domingues R (2007). Comparative genomic hybridization, BRAF, RAS, RET, and oligo-array analysis in aneuploid papillary thyroid carcinomas.. Oncol Rep.

[16] Wreesmann VB, Sieczka EM, Socci ND, Hezel M, Belbin TJ (2004). Genome-wide profiling of papillary thyroid cancer identifies MUC1 as an independent prognostic marker.. Cancer Res.

[17] Wreesmann VB, Ghossein RA, Patel SG, Harris CP, Schnaser EA (2002). Genome-wide appraisal of thyroid cancer progression.. Am J Pathol.

[18] Unger K, Malisch E, Thomas G, Braselmann H, Walch A (2008). Array CGH demonstrates characteristic aberration signatures in human papillary thyroid carcinomas governed by RET/PTC.. Oncogene.

[19] Finn S, Smyth P, O’Regan E, Cahill S, Toner M (2007). Low-level genomic instability is a feature of papillary thyroid carcinoma: an array comparative genomic hybridization study of laser capture microdissected papillary thyroid carcinoma tumors and clonal cell lines.. Arch Pathol Lab Med.

[20] Soh J, Okumura N, Lockwood WW, Yamamoto H, Shigematsu H (2009). Oncogene mutations, copy number gains and mutant allele specific imbalance (MASI) frequently occur together in tumor cells.. PLoS One.

[21] Tuna M, Knuutila S, Mills GB (2009). Uniparental disomy in cancer.. Trends Mol Med.

[22] Makishima H, Maciejewski JP (2011). Pathogenesis and consequences of uniparental disomy in cancer.. Clin Cancer Res.

[23] Stein L, Rothschild J, Luce J, Cowell JK, Thomas G (2010). Copy number and gene expression alterations in radiation-induced papillary thyroid carcinoma from chernobyl pediatric patients.. Thyroid.

[24] Astolfi A, Nannini M, Pantaleo MA, Di Battista M, Heinrich MC (2010). A molecular portrait of gastrointestinal stromal tumors: an integrative analysis of gene expression profiling and high-resolution genomic copy number.. Lab Invest.

[25] Nannya Y, Sanada M, Nakazaki K, Hosoya N, Wang L (2005). A robust algorithm for copy number detection using high-density oligonucleotide single nucleotide polymorphism genotyping arrays.. Cancer Res.

[26] Yamamoto G, Nannya Y, Kato M, Sanada M, Levine RL (2007). Highly sensitive method for genomewide detection of allelic composition in nonpaired, primary tumor specimens by use of affymetrix single-nucleotide-polymorphism genotyping microarrays.. Am J Hum Genet.

[27] Tiu RV, Gondek LP, O’Keefe CL, Huh J, Sekeres MA (2009). New lesions detected by single nucleotide polymorphism array-based chromosomal analysis have important clinical impact in acute myeloid leukemia.. J Clin Oncol.

[28] O’Keefe C, McDevitt MA, Maciejewski JP (2010). Copy neutral loss of heterozygosity: a novel chromosomal lesion in myeloid malignancies.. Blood.

[29] Dunbar AJ, Gondek LP, O’Keefe CL, Makishima H, Rataul MS (2008). 250 K single nucleotide polymorphism array karyotyping identifies acquired uniparental disomy and homozygous mutations, including novel missense substitutions of c-Cbl, in myeloid malignancies.. Cancer Res.

[30] Higgins ME, Claremont M, Major JE, Sander C, Lash AE (2007). CancerGenes: a gene selection resource for cancer genome projects.. Nucleic Acids Res.

[31] Kumagai A, Namba H, Akanov Z, Saenko VA, Meirmanov S (2007). Clinical implications of pre-operative rapid BRAF analysis for papillary thyroid cancer.. Endocr J.

[32] Nakayama H, Yoshida A, Nakamura Y, Hayashi H, Miyagi Y (2007). Clinical significance of BRAF (V600E) mutation and Ki-67 labeling index in papillary thyroid carcinomas.. Anticancer Res.

[33] Watanabe R, Hayashi Y, Sassa M, Kikumori T, Imai T (2009). Possible involvement of BRAFV600E in altered gene expression in papillary thyroid cancer.. Endocr J.

[34] Zuo H, Nakamura Y, Yasuoka H, Zhang P, Nakamura M (2007). Lack of association between BRAF V600E mutation and mitogen-activated protein kinase activation in papillary thyroid carcinoma.. Pathol Int.

[35] Feldt-Rasmussen U (2001). Iodine and Cancer.. Thyroid.

[36] Williams ED, Abrosimov A, Bogdanova T, Demidchik EP, Ito M (2008). Morphologic characteristics of Chernobyl-related childhood papillary thyroid carcinomas are independent of radiation exposure but vary with iodine intake.. Thyroid.

[37] Guan H, Ji M, Bao R, Yu H, Wang Y (2009). Association of high iodine intake with the T1799A BRAF mutation in papillary thyroid cancer.. J Clin Endocrinol Metab.

[38] Ito Y, Yoshida H, Maruo R, Morita S, Takano T (2009). BRAF mutation in papillary thyroid carcinoma in a Japanese population: its lack of correlation with high-risk clinicopathological features and disease-free survival of patients.. Endocr J.

[39] Cybulski C, Gorski B, Huzarski T, Masojc B, Mierzejewski M (2004). CHEK2 is a multiorgan cancer susceptibility gene.. Am J Hum Genet.

[40] Hsu FF, Lin TY, Chen JY, Shieh SY (2010). p53-Mediated transactivation of LIMK2b links actin dynamics to cell cycle checkpoint control.. Oncogene.

[41] Anania MC, Sensi M, Radaelli E, Miranda C, Vizioli MG (2011). TIMP3 regulates migration, invasion and in vivo tumorigenicity of thyroid tumor cells.. Oncogene.

[42] Viglietto G, Amodio N, Malanga D, Scrima M, De Marco C (2011). Contribution of PKB/AKT signaling to thyroid cancer.. Frontiers in Bioscience.

[43] Xing M (2010). Genetic alterations in the phosphatidylinositol-3 kinase/Akt pathway in thyroid cancer.. Thyroid.

[44] Zuo H, Gandhi M, Edreira MM, Hochbaum D, Nimgaonkar VL (2010). Downregulation of Rap1GAP through epigenetic silencing and loss of heterozygosity promotes invasion and progression of thyroid tumors.. Cancer Research.

[45] Nellore A, Paziana K, Ma C, Tsygankova OM, Wang Y (2009). Loss of Rap1GAP in papillary thyroid cancer.. Journal of Clinical Endocrinology and Metabolism.

[46] Siironen P, Ristimaki A, Narko K, Nordling S, Louhimo J (2006). VEGF-C and COX-2 expression in papillary thyroid cancer.. Endocr Relat Cancer.

[47] Hung CJ, Ginzinger DG, Zarnegar R, Kanauchi H, Wong MG (2003). Expression of vascular endothelial growth factor-C in benign and malignant thyroid tumors.. Journal of Clinical Endocrinology and Metabolism.

