# Supplementary figures and images for: Copy Number Alteration and Uniparental Disomy Analysis Categorizes Japanese Papillary Thyroid Carcinomas into Distinct Groups
Source: PLoS One. 2012 Apr 30;7(4):e36063. doi: 10.1371/journal.pone.0036063 (PMC3340412; doi:10.1371/journal.pone.0036063)

Supplemental figure S1

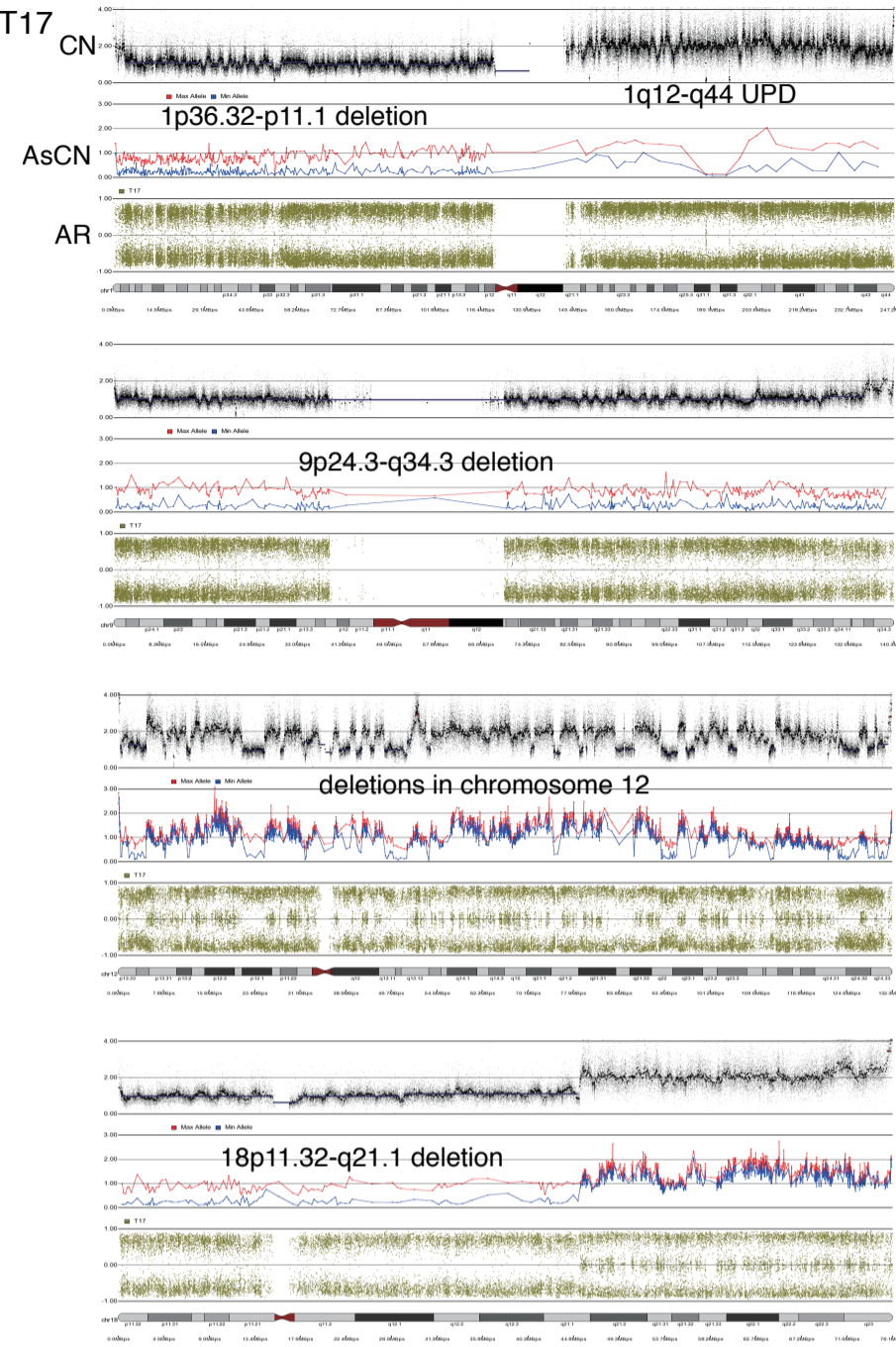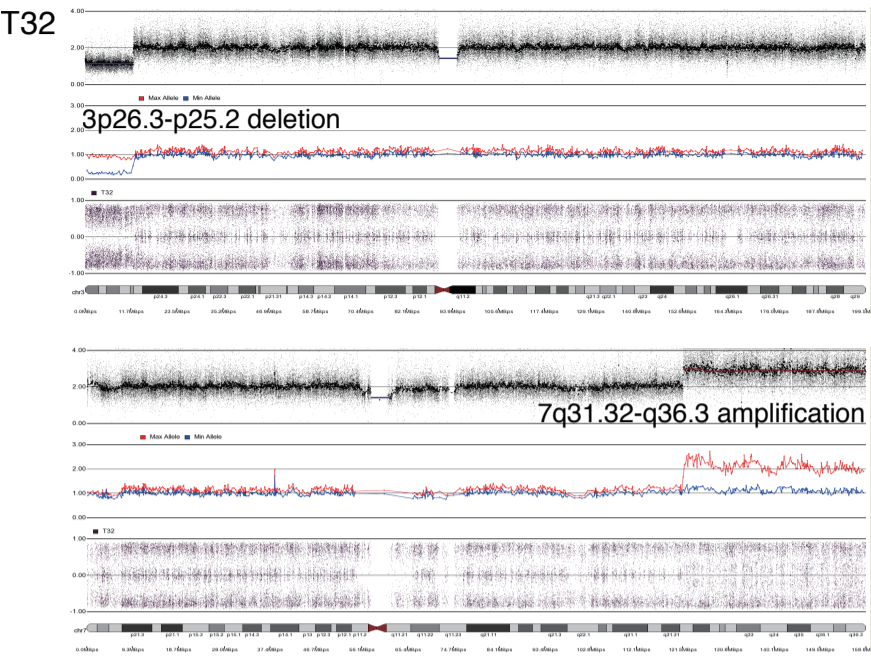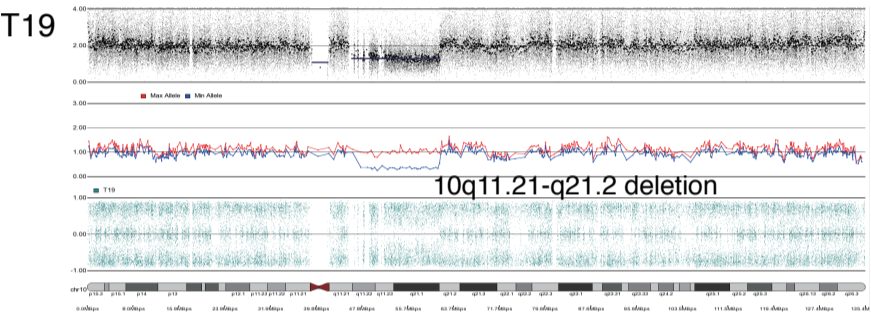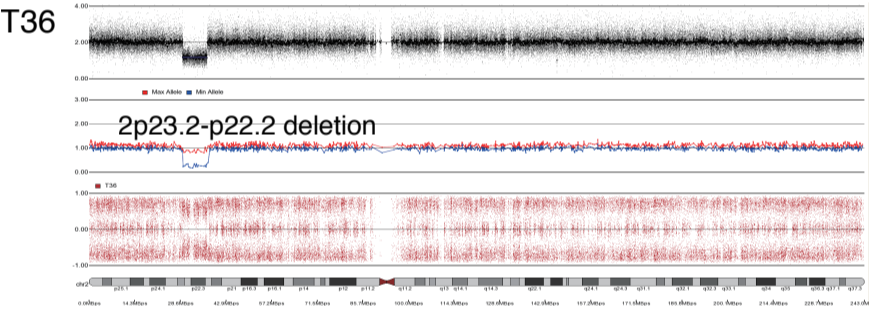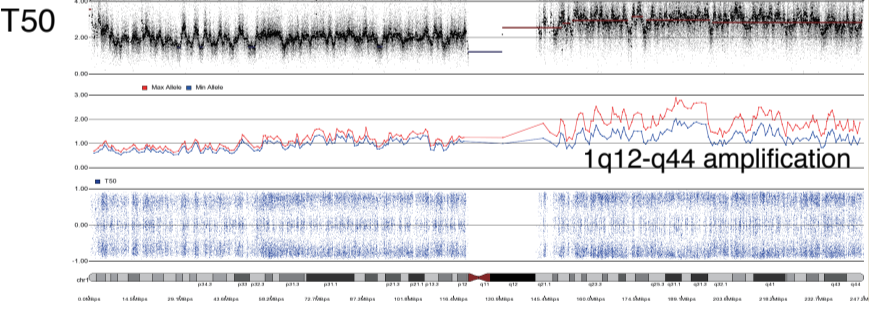

Chromosome 22 - deletion

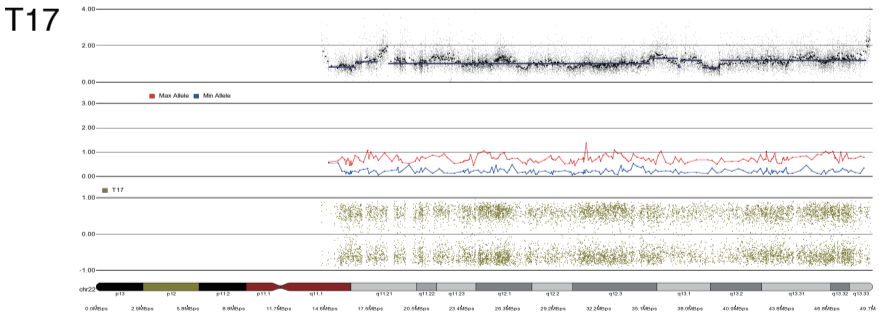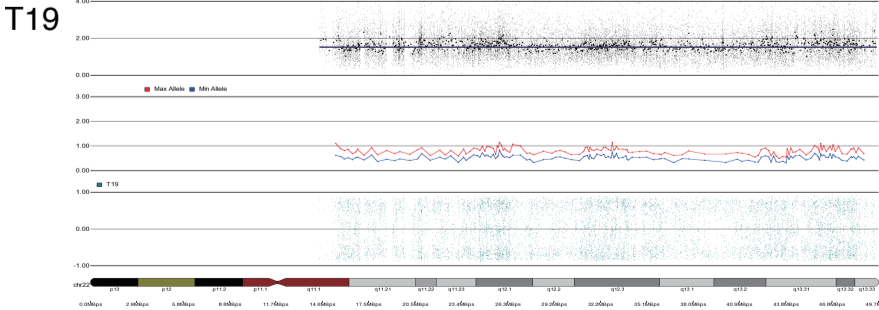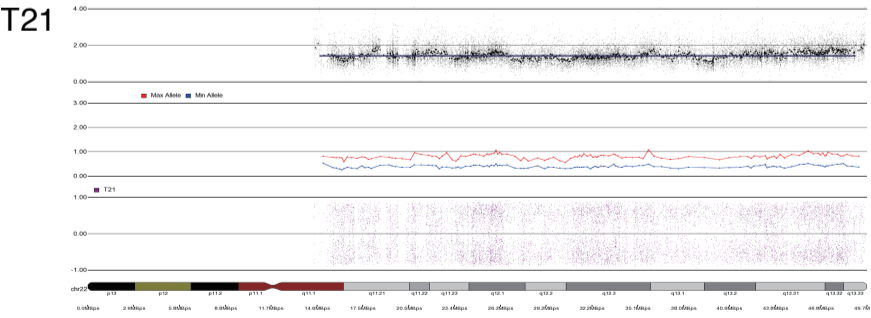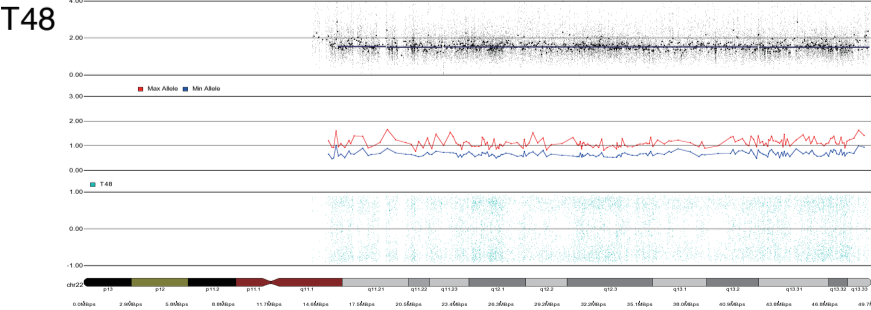

Supplement: Figure S1 — CNA regions identified using PartekGS software. CN: smoothed copy number is plotted, AsCN: allele-specific copy number analysis in which each allele is plotted separately, AR: allele ratio pattern. (PDF) [file pone.0036063.s001.pdf]

Supplemental figure S2

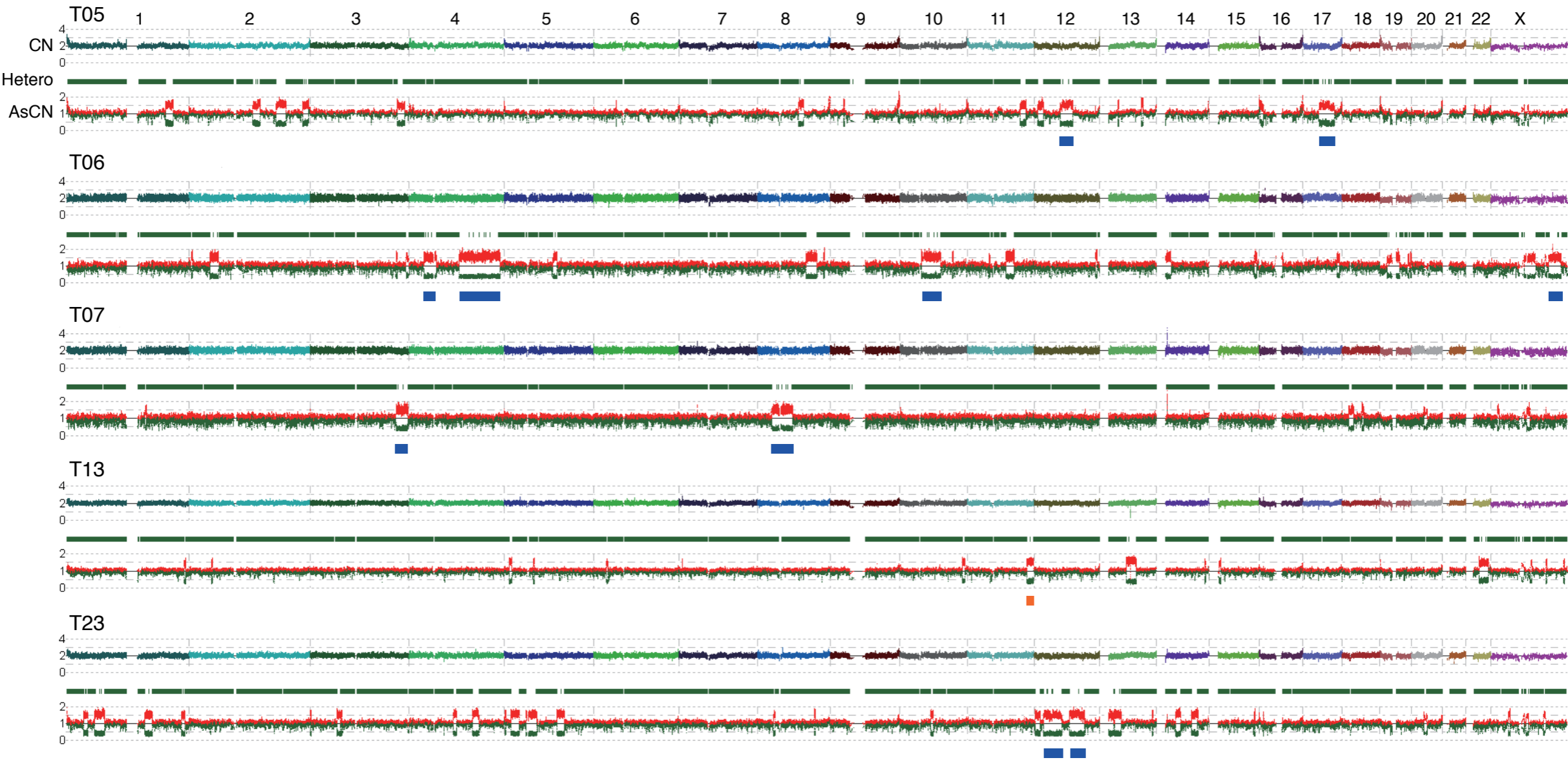

Supplement: Figure S2 — Whole-genome view of 5 PTC cases with UPD generated by CNAG/AsCNAR. CN: linear copy number smoothed over 10 SNPs is plotted in chromosomal order along the horizontal axis (chromosome 1 on the left, chromosome 22 on the right), Hetero: green bars indicate heterozygous call, AsCN: allele-specific copy number analysis in which each allele is plotted separately. Blue box represents interstitial UPD (≥24.6 Mb); Orange box represents telomeric UPD. (PDF) [file pone.0036063.s002.pdf]
