# Supplementary material for: Copy Number Alteration and Uniparental Disomy Analysis Categorizes Japanese Papillary Thyroid Carcinomas into Distinct Groups
Source: PLoS One. 2012 Apr 30;7(4):e36063. doi: 10.1371/journal.pone.0036063 (PMC3340412; doi:10.1371/journal.pone.0036063)
Supplement: Table S1 — Primer sequences. (PDF) [file pone.0036063.s003.pdf]

Supplemental Table S1. Primer sequences

Gene	Codon	Primer sequence	Annealing Temperature (℃)	Amplicon size (bp)
BRAF	600	5'-ACATACTTATTGACTCTAAGAGGAAAGATGAA-3'<br>5'-GATTTTTGTGAATACTGGGAACTATGA-3'	60	400
H-RAS	12/13	5'-AGCAGGGCCCTCCTTGGCAG-3'<br>5'-CAGCCAGCCCTATCCTGGCTG-3'	65	261
	61	5'-CAGGGAGAGGCTGGCTGTGTG-3'<br>5'-CCACCTGTGCGGCGTGGGCT-3'	65	298
K-RAS	12/13	5'-GGTACTGGTGGAGTATTTGATAGT-3'<br>5'-CTCATGAAAATGGTCAGAGAAACCT-3'	60	290
	61	5'-GGTGCACTGTAATAATCCAGACTG-3'<br>5'-CTATAATTACTCCTTAATGTCAGCTT-3'	60	268
N-RAS	12/13	5'-CACACTAGGGTTTTCATTTCCATTG-3'<br>5'-GGTAAAGATGATCCGACAAGTGAG-3'	63	283
	61	5'-TTGAACTTCCCTCCCTCCCTGC-3'<br>5'-AGCTCTATCTTCCCTAGTGTGGTAA-3'	65	316
RET/PTC1		5'-GCCTGGAGGACCTCACCAA-3'<br>5'-CTCTGCCTTTCAGATGGAA-3'	56	255
RET/PTC3		5'-ACCTGCCAGTGGTTATCAAGC-3'<br>5'-TTCGCCTTCTCCTAGAGTTTTTCC-3'	59	150
