# Supplementary material for: Copy Number Alteration and Uniparental Disomy Analysis Categorizes Japanese Papillary Thyroid Carcinomas into Distinct Groups
Source: PLoS One. 2012 Apr 30;7(4):e36063. doi: 10.1371/journal.pone.0036063 (PMC3340412; doi:10.1371/journal.pone.0036063)
Supplement: Table S2 — Chromosomal aberrations in Japanese PTCs. (PDF) [file pone.0036063.s004.pdf]

Supplemental Table S2. Chromosomal aberrations in Japanese PTCs

Case	cytoband	chr	start	end	length (bp)	Genomic alteration	Group
T13	11q23.3 - 11q25	11	120,599,042	134,449,982	13,850,941	Telomere UPD	*BRAF+*
T13	13q21.1 - 13q22.2	13	53,937,874	74,333,453	20,395,580	Interstitial UPD <24.6Mb	*BRAF+*
T13	22q12.1 - 22q13.31	22	25,968,662	44,398,349	18,429,688	Interstitial UPD <24.6Mb	*BRAF+*
T15	16p13.11 - 16p12.1	16	15,406,308	25,963,493	10,557,186	Interstitial UPD <24.6Mb	*BRAF+*
T17	1p36.32 - 1p11.1	1	3,734,952	121,184,515	117,449,564	Deletion	*BRAF+*
T17	1q12 - 1q44	1	141,510,591	247,165,315	105,654,725	Interstitial UPD ≥24.6 Mb	*BRAF+*
T17	9p24.3 - 9q34.3	9	36,587	140,051,485	140,014,899	Deletion	*BRAF+*
T17	12p13.33 - 12p13.32	12	1,735,795	4,846,088	3,110,294	Deletion	*BRAF+*
T17	12p13.2	12	11,663,693	12,411,391	747,699	Deletion	*BRAF+*
T17	12p12.1	12	21,211,759	25,041,297	3,829,539	Deletion	*BRAF+*
T17	12p11.22	12	27,727,113	28,376,271	649,159	Deletion	*BRAF+*
T17	12p11.21 - 12p11.1	12	31,414,767	33,274,165	1,859,399	Deletion	*BRAF+*
T17	12q12	12	37,759,814	39,679,272	1,919,459	Deletion	*BRAF+*
T17	12q12	12	40,683,571	41,714,348	1,030,778	Deletion	*BRAF+*
T17	12q12	12	43,066,755	43,804,551	737,797	Deletion	*BRAF+*
T17	12q13.11 - 12q13.13	12	45,516,553	50,097,565	4,581,013	Deletion	*BRAF+*
T17	12q13.13	12	50,799,502	51,516,034	716,533	Amplification	*BRAF+*
T17	12q13.13 - 12q13.2	12	52,693,168	53,459,334	766,167	Deletion	*BRAF+*
T17	12q21.2	12	74,456,393	75,555,293	1,098,901	Deletion	*BRAF+*
T17	12q21.31	12	78,845,628	79,524,296	678,669	Deletion	*BRAF+*
T17	12q21.31	12	80,049,860	80,204,711	154,852	Deletion	*BRAF+*
T17	12q21.31 - 12q21.32	12	85,010,138	86,458,591	1,448,454	Deletion	*BRAF+*
T17	12q21.32 - 12q21.33	12	86,626,715	87,948,168	1,321,454	Deletion	*BRAF+*
T17	12q21.33	12	88,087,657	88,330,873	243,217	Deletion	*BRAF+*
T17	12q22 - 12q23.1	12	92,803,212	95,656,784	2,853,573	Deletion	*BRAF+*
T17	12q23.1	12	97,054,890	98,577,685	1,522,796	Deletion	*BRAF+*
T17	12q23.3	12	104,304,452	105,804,313	1,499,862	Deletion	*BRAF+*
T17	12q24.13	12	111,247,474	112,401,485	1,154,012	Deletion	*BRAF+*
T17	12q24.31 - 12q24.32	12	123,098,750	126,680,137	3,581,388	Deletion	*BRAF+*
T17	12q24.33	12	129,770,792	131,722,958	1,952,167	Deletion	*BRAF+*
T17	18p11.32 - 18q21.1	18	190,416	45,359,569	45,169,154	Deletion	*BRAF+*
T17	22q11.1 - 22q13.33	22	14,857,285	49,286,917	34,429,633	Deletion	*BRAF+*
T21	22q11.1 - 22q13.33	22	14,780,993	48,960,610	34,179,618	Deletion	*BRAF+*
T23	1p32.2 - 1p31.1	1	56,903,176	76,557,224	19,654,049	Interstitial UPD <24.6Mb	*BRAF+*
T23	1q23.2 - 1q25.1	1	158,056,061	173,439,162	15,383,102	Interstitial UPD <24.6Mb	*BRAF+*
T23	4q28.1 - 4q31.1	4	127,417,835	140,753,568	13,335,734	Interstitial UPD <24.6Mb	*BRAF+*
T23	5p15.2 - 5p13.3	5	13,782,714	30,975,726	17,193,013	Interstitial UPD <24.6Mb	*BRAF+*
T23	5p12 - 5q12.3	5	44,428,734	65,946,072	21,517,339	Interstitial UPD <24.6Mb	*BRAF+*
T23	5q21.3 - 5q23.1	5	106,877,336	121,216,508	14,339,173	Interstitial UPD <24.6Mb	*BRAF+*
T23	12p13.33 - 12p13.1	12	2,049,021	12,637,852	10,588,832	Interstitial UPD <24.6Mb	*BRAF+*
T23	12p12.2 - 12q13.3	12	20,131,592	56,232,127	36,100,536	Interstitial UPD ≥24.6 Mb	*BRAF+*
T23	12q21.1 - 12q23.2	12	71,297,634	101,654,192	30,356,559	Interstitial UPD ≥24.6 Mb	*BRAF+*
T23	13q11 - 13q12.3	13	18,113,674	28,938,001	10,824,328	Interstitial UPD <24.6Mb	*BRAF+*
T23	13q12.3 - 13q14.11	13	30,025,975	43,134,266	13,108,292	Interstitial UPD <24.6Mb	*BRAF+*
T23	14q24.2 - 14q31.1	14	70,422,251	82,328,673	11,906,423	Interstitial UPD <24.6Mb	*BRAF+*
T05	1q32.1 - 1q41	1	199,437,906	215,295,755	15,857,850	Interstitial UPD <24.6Mb	*wt*
T05	2q14.3 - 2q22.1	2	128,122,391	142,212,677	14,090,287	Interstitial UPD <24.6Mb	*wt*
T05	2q31.1 - 2q32.3	2	174,419,674	194,612,336	20,192,663	Interstitial UPD <24.6Mb	*wt*
T05	2q36.3 - 2q37.3	2	227,747,402	239,939,302	12,191,901	Interstitial UPD <24.6Mb	*wt*
T05	3q26.31 - 3q28	3	175,896,645	190,526,993	14,630,349	Interstitial UPD <24.6Mb	*wt*
T05	8q21.13 - 8q21.3	8	82,316,787	93,459,316	11,142,530	Interstitial UPD <24.6Mb	*wt*
T05	11q22.3 - 11q23.3	11	107,084,771	119,208,981	12,124,211	Interstitial UPD <24.6Mb	*wt*
T05	12p13.31 - 12p12.3	12	8,007,288	19,688,787	11,681,500	Interstitial UPD <24.6Mb	*wt*
T05	12q13.2 - 12q21.2	12	53,197,104	78,294,659	25,097,556	Interstitial UPD ≥24.6 Mb	*wt*
T05	17q12 - 17q24.2	17	32,095,746	63,137,043	31,041,298	Interstitial UPD ≥24.6 Mb	*wt*
T06	2p21 - 2p16.1	2	41,957,481	59,226,475	17,268,995	Interstitial UPD <24.6Mb	*wt*
T06	4p15.1 - 4q12	4	29,575,338	55,005,069	25,429,732	Interstitial UPD ≥24.6 Mb	*wt*
T06	4q23 - 4q35.1	4	101,448,476	182,929,392	81,480,917	Interstitial UPD ≥24.6 Mb	*wt*
T06	8q22.1 - 8q24.11	8	97,183,808	119,185,600	22,001,793	Interstitial UPD <24.6Mb	*wt*
T06	10q11.21 - 10q23.1	10	43,423,622	82,409,749	38,986,128	Interstitial UPD ≥24.6 Mb	*wt*
T06	11q14.1 - 11q21	11	78,592,389	95,070,831	16,478,443	Interstitial UPD <24.6Mb	*wt*
T06	14q11.2 - 14q12	14	19,281,484	29,648,517	10,367,034	Interstitial UPD <24.6Mb	*wt*
T06	Xq13.1 - Xq21.31	X	68,398,493	90,023,794	21,625,302	Interstitial UPD <24.6Mb	*wt*
T06	Xq24 - Xq27.3	X	116,906,013	142,428,335	25,522,323	Interstitial UPD ≥24.6 Mb	*wt*
T32	3p26.3 - 3p25.2	3	35,345	12,306,320	12,270,976	Deletion	*wt*
T32	7q31.32 - 7q36.3	7	121,540,345	158,819,766	37,279,422	Amplification	*wt*
T36	2p23.2 - 2p22.2	2	29,300,617	36,995,231	7,694,615	Deletion	*wt*
T48	22q11.1 - 22q13.33	22	15,986,921	49,581,322	33,594,402	Deletion	*wt*
T50	1q12 - 1q44	1	141,510,591	247,191,012	105,680,422	Amplification	*wt*
T07	3q26.31 - 3q29	3	173,269,668	197,898,556	24,628,889	Interstitial UPD ≥24.6 Mb	*wt*
T07	8p21.1 - 8q13.3	8	29,271,088	71,028,133	41,757,046	Interstitial UPD ≥24.6 Mb	*wt*
T19	10q11.21 - 10q21.2	10	45,939,485	61,302,814	15,363,330	Deletion	*RET/PTC1*
T19	22q11.1 - 22q13.33	22	14,432,528	49,518,269	35,085,742	Deletion	*RET/PTC1*
