# Supplementary material for: Copy Number Alteration and Uniparental Disomy Analysis Categorizes Japanese Papillary Thyroid Carcinomas into Distinct Groups
Source: PLoS One. 2012 Apr 30;7(4):e36063. doi: 10.1371/journal.pone.0036063 (PMC3340412; doi:10.1371/journal.pone.0036063)
Supplement: Table S3 — CancerGenes located in recurrent chromosome 22q deletion. (PDF) [file pone.0036063.s005.pdf]

Supplemental Table S3. CancerGenes located in recurrent chromosome 22q deletion

Gene Symbol	Gene Name	GO Categories	Sources
PRODH	proline dehydrogenase (oxidase) 1	cellular_component;glutamate biosynthetic process;induction of apoptosis by oxidative stress;mitochondrion;molecular_function;oxidoreductase activity;proline catabolic process;proline dehydrogenase activity	Stability, Tumor Suppressor
TSSK2	testis-specific serine kinase 2	ATP binding;cell differentiation;magnesium ion binding;multicellular organismal development;nucleotide binding;protein amino acid phosphorylation;protein binding;protein serine/threonine kinase activity;spermatogenesis;transferase activity	Protein Kinase
CLTCL1	clathrin, heavy chain-like 1	anatomical structure morphogenesis;binding;clathrin vesicle coat;coated pit;receptor-mediated endocytosis;signal transducer activity	Futreal et al 2004, Mitelman 2000, Translocation
GP1BB	septin 5	cell cycle;cytokinesis;GTP binding;GTPase activity;nucleotide binding;plasma membrane;protein binding;regulation of exocytosis;structural molecule activity;synaptic vesicle;synaptic vesicle targeting	Futreal et al 2004, Mitelman 2000, Translocation
COMT	catechol-O-methyltransferase	catechol O-methyltransferase activity;catecholamine metabolic process;dopamine catabolic process;integral to membrane;magnesium ion binding;membrane;microsome;O-methyltransferase activity;protein binding;soluble fraction;transferase activity	Stability
CRKL	v-crk sarcoma virus CT10 oncogene homolog (avian)-like	anterior/posterior pattern formation;blood vessel development;cell motility;heart development;intracellular signaling cascade;JNK cascade;organ morphogenesis;parathyroid gland development;pattern specification process;protein amino acid phosphorylation;protein binding;protein-tyrosine kinase activity;Ras protein signal transduction;SH3/SH2 adaptor activity;thymus development	EGFR1, Kit Receptor, Protein Kinase, Entrez Query:Oncogene
UBE2L3	ubiquitin-conjugating enzyme E2L 3	enzyme binding;ligase activity;protein modification process;small conjugating protein ligase activity;ubiquitin cycle;ubiquitin ligase complex;ubiquitin-dependent protein catabolic process;ubiquitin-protein ligase activity	TGFBR
MAPK1	mitogen-activated protein kinase 1	ATP binding;cell cycle;chemotaxis;cytoplasm;cytosine metabolic process;induction of apoptosis;MAP kinase activity;MAPKKK cascade;negative regulation of cell differentiation;nucleotide binding;nucleus;organ morphogenesis;phosphotyrosine binding;protein amino acid phosphorylation;protein binding;protein serine/threonine kinase activity;response to DNA damage stimulus;signal transduction;synaptic transmission;transferase activity	Protein Kinase, Stability, Tumor Suppressor, Entrez Query:Oncogene
TOP3B	topoisomerase (DNA) III beta	chromosome organization and biogenesis (sensu Eukaryota);chromosome segregation;condensed chromosome;DNA modification;DNA topoisomerase type I activity;DNA topological change;DNA unwinding during replication;nucleus;protein binding	Stability
RAB36	RAB36, member RAS oncogene family	Golgi apparatus;GTP binding;GTPase activity;nucleotide binding;protein transport;small GTPase mediated signal transduction	Entrez Query:Oncogene
BCR	breakpoint cluster region	cellular_component;GTPase activator activity;guanyl-nucleotide exchange factor activity;intracellular;intracellular signaling cascade;kinase activity;protein amino acid phosphorylation;protein serine/threonine kinase activity;regulation of Rho protein signal transduction;Rho guanyl-nucleotide exchange factor activity;signal transduction;transferase activity	Futreal et al 2004, Mitelman 2000, Tumor Suppressor, Entrez Query:Oncogene, Translocation
RGL4	-	guanyl-nucleotide exchange factor activity;intracellular;small GTPase mediated signal transduction	Entrez Query:Oncogene
MMP11	matrix metallopeptidase 11 (stromelysin 3)	anatomical structure morphogenesis;calcium ion binding;collagen catabolic process;multicellular organismal development;proteinaceous extracellular matrix;proteolysis;stromelysin 3 activity;zinc ion binding	Stability
SMARCB1	SWI/SNF related, matrix associated, actin dependent regulator of chromatin, subfamily b, member 1	cell cycle;chromatin remodeling;DNA integration;negative regulation of progression through cell cycle;nuclear chromosome;nucleoplasm;nucleus;protein binding;regulation of transcription from RNA polymerase II promoter;retroviral genome replication;SWI/SNF complex;transcription	TNF_alpha_NF_kB, Futreal et al 2004, Tumor Suppressor, Entrez Query:Oncogene, Frameshift mutation, Large deletion, Nonsense mutation, Splicing mutation
MIF	macrophage migration inhibitory factor (glycosylation-inhibiting factor)	cell proliferation;cell surface receptor linked signal transduction;cytokine activity;extracellular region;extracellular space;inflammatory response;isomerase activity;negative regulation of apoptosis;phenylpyruvate tautomerase activity;prostaglandin biosynthetic process;protein binding;regulation of macrophage activation	Protein Kinase, Stability
GSTT1	glutathione S-transferase theta 1	glutathione transferase activity;response to stress;transferase activity	Stability
ADORA2A	adenosine A2a receptor	A2A adenosine receptor activity, G-protein coupled;adenylate cyclase activation;apoptosis;blood coagulation;cAMP biosynthetic process;cell-cell signaling;cellular defense response;central nervous system development;circulation;G-protein coupled receptor protein signaling pathway;G-protein signaling, coupled to cAMP nucleotide second messenger;gastric inhibitory peptide receptor activity;inflammatory response;integral to membrane;integral to plasma membrane;membrane;membrane fraction;neurotransmitter transpo	Stability
ADRBK2	adrenergic, beta, receptor kinase 2	ATP binding;G-protein coupled receptor kinase activity;nucleotide binding;protein amino acid phosphorylation;signal transducer activity;signal transduction;transferase activity	Protein Kinase
MYO18B	myosin XVIIIB	actin binding;ATP binding;motor activity;nucleotide binding;nucleus;unconventional myosin complex	Tumor Suppressor
CRYBB1	crystallin, beta B1	structural constituent of eye lens;visual perception	Stability
MN1	meningioma (disrupted in balanced translocation) 1	biological_process;cell cycle;cellular_component;molecular_function;negative regulation of progression through cell cycle	Futreal et al 2004, Mitelman 2000, Tumor Suppressor, Entrez Query:Oncogene, Translocation
CHEK2	CHK2 checkpoint homolog (S. pombe)	ATP binding;cell cycle;DNA damage checkpoint;DNA damage response, signal transduction resulting in induction of apoptosis;magnesium ion binding;nucleotide binding;nucleus;PML body;protein amino acid phosphorylation;protein binding;protein serine/threonine kinase activity;transferase activity	Futreal et al 2004, Protein Kinase, Stability, Tumor Suppressor, Frameshift mutation

EWSR1	Ewing sarcoma breakpoint region 1	calmodulin binding;intracellular;metal ion binding;nucleic acid binding;nucleotide binding;nucleus;regulation of transcription, DNA-dependent;RNA binding;transcription;zinc ion binding	Futreal et al 2004, Mitelman 2000, Vogelstein and Kinzler 2004, Entrez Query:Oncogene, Translocation
RASL10A	RAS-like, family 10, member A	GTP binding;GTPase activity;intracellular;membrane;nucleotide binding;small GTPase mediated signal transduction	Tumor Suppressor
NF2	neurofibromin 2 (bilateral acoustic neuroma)	cell cycle;cytoplasm;cytoskeletal protein binding;cytoskeleton;negative regulation of cell proliferation;negative regulation of progression through cell cycle;nucleus;plasma membrane;sensory perception of sound;structural molecule activity	Futreal et al 2004, Hahn and Weinberg 2002, Vogelstein and Kinzler 2004, Protein Kinase, Tumor Suppressor, Frameshift mutation, Large deletion, Missense mutation, Nonsense mutation, Splicing mutation
LIF	leukemia inhibitory factor (cholinergic differentiation factor)	cell surface receptor linked signal transduction;cell-cell signaling;cytokine activity;extracellular region;extracellular space;growth factor activity;immune response;leukemia inhibitory factor receptor binding;multicellular organismal development;oncostatin-M receptor binding;positive regulation of cell proliferation	Tumor Suppressor
OSM	oncostatin M	apoptosis;behavioral response to pain;cell proliferation;cell-cell signaling;cytokine activity;extracellular region;extracellular space;immune response;multicellular organismal development;negative regulation of cell proliferation;oncostatin-M receptor binding;peripheral nervous system development;regulation of cell growth;response to heat;tyrosine phosphorylation of Stat1 protein;tyrosine phosphorylation of Stat3 protein;tyrosine phosphorylation of Stat5 protein	Tumor Suppressor
SEC14L2	SEC14-like 2 (S. cerevisiae)	cytoplasm;integral to membrane;intracellular;nucleus;phospholipid binding;positive regulation of transcription, DNA-dependent;protein carrier activity;regulation of cholesterol biosynthetic process;transcription;transcriptional activator activity;transport;transporter activity;vitamin E binding	Tumor Suppressor
PES1	pescadillo homolog 1, containing BRCT domain (zebrafish)	anatomical structure morphogenesis;cell proliferation;intracellular;nucleolus;nucleus;protein binding	Stability, Tumor Suppressor
DUSP18	dual specificity phosphatase 18	hydrolase activity;nucleus;protein amino acid dephosphorylation;protein tyrosine phosphatase activity;protein tyrosine/serine/threonine phosphatase activity	Phosphatase
LIMK2	LIM domain kinase 2	ATP binding;cytoplasm;metal ion binding;nucleotide binding;nucleus;protein amino acid phosphorylation;protein binding;protein kinase activity;protein serine/threonine kinase activity;transferase activity;zinc ion binding	Protein Kinase, Tyrosine Kinase
PATZ1	POZ (BTB) and AT hook containing zinc finger 1	DNA binding;intracellular;metal ion binding;nucleus;protein binding;regulation of transcription, DNA-dependent;transcription;transcriptional repressor activity;zinc ion binding	Androgen Receptor, Futreal et al 2004, Mitelman 2000, Entrez Query:Oncogene, Translocation
DRG1	developmentally regulated GTP binding protein 1	GTP binding;intracellular;multicellular organismal development;nucleotide binding;transcription;transcription factor binding	Tumor Suppressor
YWHAH	tyrosine 3-monooxygenase/tryptophan 5-monooxygenase activation protein, eta polypeptide	actin binding;cytoplasm;cytoskeleton organization and biogenesis;glucocorticoid catabolic process;glucocorticoid receptor binding;glucocorticoid receptor signaling pathway;insulin-like growth factor receptor binding;intracellular protein transport;negative regulation of apoptosis;negative regulation of dendrite morphogenesis;negative regulation of protein kinase activity;positive regulation of transcription;protein domain specific binding;protein kinase C inhibitor activity;regulation of mitosis;regulation	Alpha6Beta4 Integrin, TNF_alpha_NF_kB, Tumor Suppressor
FBXO7	F-box protein 7	protein binding;regulation of protein stability;ubiquitin cycle;ubiquitin ligase complex;ubiquitin-dependent protein catabolic process;ubiquitin-protein ligase activity	Stability
TIMP3	TIMP metallopeptidase inhibitor 3 (Sorsby fundus dystrophy, pseudoinflammatory)	basement membrane;enzyme inhibitor activity;induction of apoptosis by extracellular signals;metalloendopeptidase inhibitor activity;molecular_function;proteinaceous extracellular matrix;response to stimulus;transmembrane receptor protein tyrosine kinase signaling pathway;visual perception	Tumor Suppressor, Entrez Query:Oncogene
TOM1	target of myb1 (chicken)	cytosol;early endosome;endocytosis;endosome;endosome transport;intracellular;intracellular protein transport;lysosome;membrane;protein binding	Entrez Query:Oncogene
HMOX1	heme oxygenase (decycling) 1	endoplasmic reticulum;heme oxidation;heme oxygenase (decyclizing) activity;iron ion binding;membrane fraction;metal ion binding;microsome;oxidoreductase activity;positive regulation of I-kappaB kinase/NF-kappaB cascade;signal transducer activity	Protein Kinase
MCM5	minichromosome maintenance complex component 5	ATP binding;DNA binding;DNA replication;DNA replication initiation;DNA-dependent ATPase activity;nucleotide binding;nucleus;protein binding;regulation of progression through cell cycle;regulation of transcription, DNA-dependent;transcription	TNF_alpha_NF_kB
MYH9	myosin, heavy chain 9, non-muscle	actin cytoskeleton reorganization;actin filament binding;actin filament-based movement;angiogenesis;ATP binding;ATPase activity;blood vessel endothelial cell migration;calmodulin binding;cleavage furrow;contractile ring;cytokinesis;cytoplasm;cytosol;integrin complex;integrin-mediated signaling pathway;leukocyte migration;membrane protein ectodomain proteolysis;microfilament motor activity;monocyte differentiation;motor activity;myosin complex;nucleotide binding;nucleus;plasma membrane;platelet formation;pro	Futreal et al 2004, Stability, Translocation
CACNG2	calcium channel, voltage-dependent, gamma subunit 2	calcium ion binding;calcium ion transport;integral to membrane;ion transport;membrane;membrane fraction;regulation of membrane potential;structural molecule activity;tight junction;transmission of nerve impulse;voltage-gated calcium channel activity;voltage-gated calcium channel complex	Stability
CSF2RB	colony stimulating factor 2 receptor, beta, low-affinity (granulocyte-macrophage)	cytokine and chemokine mediated signaling pathway;granulocyte macrophage colony-stimulating factor receptor complex;interleukin-3 receptor activity;interleukin-5 receptor activity;membrane;receptor activity;respiratory gaseous exchange;signal transduction	Kit Receptor

MFNG	MFNG O-fucosylpeptide 3-beta-N-acetylglucosaminyltransferase	extracellular space;integral to membrane;membrane;pattern specification process;transferase activity, transferring glycosyl groups	NOTCH
POLR2F	polymerase (RNA) II (DNA directed) polypeptide F	DNA binding;DNA-directed RNA polymerase activity;nucleus;regulation of transcription;RNA polymerase complex;transcription from RNA polymerase II promoter;transferase activity	Protein Kinase
PICK1	protein interacting with PRKCA 1	ATPase activity;cytoplasm;DNA methylation during embryonic development;DNA methylation during gametogenesis;dopamine transport;ephrin receptor binding;Golgi apparatus;GTPase binding;metabotropic glutatmate receptor binding;mitochondrion;monoamine transport;multicellular organismal development;neuronal ion channel clustering;perinuclear region of cytoplasm;plasma membrane;presynaptic membrane;protein amino acid phosphorylation;protein C-terminus binding;protein homooligomerization;protein kinase C activation	Protein Kinase
MAFF	v-maf musculoaponeurotic fibrosarcoma oncogene homolog F (avian)	in utero embryonic development;nucleus;parturition;protein dimerization activity;regulation of epidermal cell differentiation;regulation of transcription, DNA-dependent;sequence-specific DNA binding;transcription;transcription factor activity;transcription from RNA polymerase II promoter	Entrez Query:Oncogene
CSNK1E	casein kinase 1, epsilon	ATP binding;casein kinase I activity;DNA repair;nucleotide binding;protein amino acid phosphorylation;protein binding;protein serine/threonine kinase activity;signal transduction;transferase activity	Wnt, Protein Kinase, Stability
DMC1	DMC1 dosage suppressor of mck1 homolog, meiosis-specific homologous recombination (yeast)	ATP binding;cell cycle;condensed nuclear chromosome;DNA binding;DNA metabolic process;DNA-dependent ATPase activity;female gamete generation;intracellular;meiosis;meiotic recombination;nucleoside-triphosphatase activity;nucleotide binding;nucleus;ovarian follicle development;spermatogenesis	Stability
CBY1	chibby homolog 1 (Drosophila)	beta-catenin binding;identical protein binding;negative regulation of transcription, DNA-dependent;negative regulation of Wnt receptor signaling pathway;nucleus;protein localization;trans-Golgi network	Tumor Suppressor
PDGFB	platelet-derived growth factor beta polypeptide (simian sarcoma viral (v-sis) oncogene homolog)	cell proliferation;extracellular region;growth factor activity;membrane;platelet-derived growth factor receptor binding;regulation of progression through cell cycle;response to wounding	Futreal et al 2004, Mitelman 2000, Vogelstein and Kinzler 2004, Entrez Query:Oncogene, Translocation
ATF4	activating transcription factor 4 (tax-responsive enhancer element B67)	amino acid metabolic process;cytoplasm;gluconeogenesis;nucleus;positive regulation of transcription from RNA polymerase II promoter;protein dimerization activity;regulation of transcription, DNA-dependent;response to stress;sequence-specific DNA binding;specific RNA polymerase II transcription factor activity;transcription;transcription factor activity;transcriptional activator activity	Stability
MKL1	megakaryoblastic leukemia (translocation) 1	actin binding;anti-apoptosis;cytoplasm;nucleic acid binding;nucleus;positive regulation of transcription;protein binding;regulation of transcription, DNA-dependent;transcription;transcription coactivator activity	Futreal et al 2004, Mitelman 2000, Translocation
ST13	suppression of tumorigenicity 13 (colon carcinoma) (Hsp70 interacting protein)	binding;cytoplasm;protein binding, bridging;protein folding	Tumor Suppressor
RBX1	ring-box 1	DNA repair;metal ion binding;nucleus;protein binding;protein catabolic process;ubiquitin cycle;zinc ion binding	NOTCH, TGFBR, Stability
EP300	E1A binding protein p300	acetyltransferase activity;apoptosis;cell cycle;heart development;histone acetylation;histone acetyltransferase activity;histone acetyltransferase complex;homeostatic process;lung development;metal ion binding;N-terminal peptidyl-lysine acetylation;nervous system development;nucleus;organ morphogenesis;positive regulation of transcription factor activity;positive regulation of transcription, DNA-dependent;protein C-terminus binding;response to hypoxia;signal transduction;somitogenesis;striated muscle develo	Androgen Receptor, Kit Receptor, NOTCH, TGFBR, Wnt, Futreal et al 2004, Mitelman 2000, Translocation
XRCC6	X-ray repair complementing defective repair in Chinese hamster cells 6 (Ku autoantigen, 70kDa)	ATP-dependent DNA helicase activity;cytoplasm;DNA binding;DNA ligation;DNA recombination;DNA-dependent protein kinase complex;double-strand break repair via nonhomologous end joining;double-stranded DNA binding;membrane fraction;nucleus;positive regulation of transcription, DNA-dependent;protein binding;transcription factor complex	Stability
BIK	BCL2-interacting killer (apoptosis-inducing)	apoptotic program;induction of apoptosis;integral to membrane;membrane;mitochondrial envelope;protein binding;regulation of apoptosis	Tumor Suppressor
PARVG	parvin, gamma	actin binding;cell adhesion;cell-matrix adhesion;cytoskeleton;protein binding	Tumor Suppressor
PRR5	proline rich 5 (renal)	GTPase activator activity;intracellular;signal transduction	Tumor Suppressor
FBLN1	fibulin 1	calcium ion binding;extracellular matrix structural constituent;extracellular region;extracellular space;proteinaceous extracellular matrix;soluble fraction	Tumor Suppressor
PPARA	peroxisome proliferator-activated receptor alpha	epidermis development;fatty acid transport;lipid metabolic process;metal ion binding;nucleus;positive regulation of fatty acid beta-oxidation;positive regulation of transcription;positive regulation of transcription from RNA polymerase II promoter;protein binding;receptor activity;regulation of fatty acid metabolic process;regulation of transcription, DNA-dependent;sequence-specific DNA binding;steroid hormone receptor activity;transcription;transcription factor activity;transcription from RNA polymerase II	Tumor Suppressor
GTSE1	G-2 and S-phase expressed 1	cytoplasmic microtubule;DNA damage response, signal transduction by p53 class mediator resulting in cell cycle arrest;G2 phase of mitotic cell cycle;microtubule-based process;molecular_function	Tumor Suppressor
CERK	ceramide kinase	calcium ion binding;ceramide kinase activity;ceramide metabolic process;diacylglycerol kinase activity;integral to membrane of membrane fraction;kinase activity;magnesium ion binding;protein kinase C activation;transferase activity	Protein Kinase
PIM3	pim-3 oncogene	ATP binding;nucleotide binding;protein amino acid phosphorylation;protein kinase activity;protein serine/threonine kinase activity;transferase activity	Protein Kinase, Entrez Query:Oncogene
PANX2	pannexin 2	gap junction;integral to membrane;membrane	Tumor Suppressor
