# Supplementary material for: Copy Number Alteration and Uniparental Disomy Analysis Categorizes Japanese Papillary Thyroid Carcinomas into Distinct Groups
Source: PLoS One. 2012 Apr 30;7(4):e36063. doi: 10.1371/journal.pone.0036063 (PMC3340412; doi:10.1371/journal.pone.0036063)
Supplement: Table S4 — RAS family/MAPK pathway genes from CancerGenes database and PTC-related genes located in the CNA/UPD region. (PDF) [file pone.0036063.s006.pdf]

**Supplemental Table S4. RAS family/MAPK pathway genes from CancerGenes database and PTC-related genes located in the CNA/UPD region**

| Cytoband | Genomic alteration | Case | Gene Symbol | Gene Name |
|---|---|---|---|---|
| 1p36.32 - 1p11.1 | Deletion | T17 | RAP1GAP | RAP1 GTPase activating protein |
| 1q12-1q44 | UPD | T17 | PTPN7 | protein tyrosine phosphatase, non-receptor type 7 |
| 1q12 - 1q44 | UPD, Amplification | T17, T50 | RAB13 | RAB13, member RAS oncogene family |
| | | | RIT1 | Ras-like without CAAX 1 |
| | | | RAB25 | RAB25, member RAS oncogene family |
| | | | RASAL2 | RAS protein activator like 2 |
| | | | RAB7L1 | RAB7, member RAS oncogene family-like 1 |
| | | | RASSF5 | Ras association (RalGDS/AF-6) domain family 5 |
| | | | RAB4A | RAB4A, member RAS oncogene family |
| | | | NTRK1 | neurotrophic tyrosine kinase, receptor, type 1 |
| | | | AKT3 | v-akt murine thymoma viral oncogene homolog 3 (protein kinase B, gamma) |
| | | | miR-181b | |
| 3q26.31-3q29 | UPD | T07 | PIK3CA | phosphoinositide-3-kinase, catalytic, alpha polypeptide |
| 4p15.1 - 4q12 | UPD | T06 | RHOH | ras homolog gene family, member H |
| | | | PDGFRA | platelet-derived growth factor receptor, alpha polypeptide |
| 4q23 - 4q35.1 | UPD | T06 | RAB33B | RAB33B, member RAS oncogene family |
| | | | GAB1 | GRB2-associated binding protein 1 |
| | | | VEGFC | vascular endothelial growth factor C |
| | | | FGF2 | fibroblast growth factor 2 (basic) |
| 7q31.32 - 7q36.3 | Amplification | T32 | BRAF | v-raf murine sarcoma viral oncogene homolog B1 |
| 8p21.1 - 8q13.3 | UPD | T07 | FGFR1 | fibroblast growth factor receptor 1 (fms-related tyrosine kinase 2, Pfeiffer syndrome) |
| | | | RAB2A | RAB2A, member RAS oncogene family |
| 9p24.3 - 9q34.3 | Deletion | T17 | RASEF | RAS and EF-hand domain containing |
| 10q11.21 - 10q23.1 | UPD | T06 | RASSF4 | Ras association (RalGDS/AF-6) domain family 4 |
| | | | RET | ret proto-oncogene |
| 12p12.2 - 12q13.3, 12q13.2 - 12q21.2, 12q21.1-12q23.2 | UPD | T23, T05 | KRAS | v-Ki-ras2 Kirsten rat sarcoma viral oncogene homolog |
| | | | SSPN | sarcospan (Kras oncogene-associated gene) |
| | | | RAB5B | RAB5B, member RAS oncogene family |
| | | | RASSF3 | Ras association (RalGDS/AF-6) domain family 3 |
| | | | RAP1B | RAP1B, member of RAS oncogene family |
| | | | RAB21 | RAB21, member RAS oncogene family |
| | | | IGFBP6 | insulin-like growth factor binding protein 6 |
| | | | DUSP6 | dual specificity phosphatase 6 |
| | | | PTPRR | protein tyrosine phosphatase, receptor type, R |
| 12q24.13 | Deletion | T17 | RASAL1 | RAS protein activator like 1 (GAP1 like) |
| 17q12 - 17q24.2 | UPD | T05 | ERBB2 | v-erb-b2 erythroblastic leukemia viral oncogene homolog 2, neuro/glioblastoma derived oncogene homolog (avian) |
| | | | NKIRAS2 | NFKB inhibitor interacting Ras-like 2 |
| | | | RAB5C | RAB5C, member RAS oncogene family |
| | | | DUSP3 | dual specificity phosphatase 3 |
| 22q11.1 - 22q13.33 | Deletion | T21, T19, T17, T48 | RASL10A | RAS-like, family 10, member A |
| Xq24 - Xq27.3 | UPD | T06 | RAB33A | RAB33A, member RAS oncogene family |
| | | | RAP2C | RAP2C, member of RAS oncogene family |
